# Emotion regulation individual therapy for adolescents with nonsuicidal self-injury disorder: a feasibility study

**DOI:** 10.1186/s12888-017-1527-4

**Published:** 2017-12-28

**Authors:** Johan Bjureberg, Hanna Sahlin, Clara Hellner, Erik Hedman-Lagerlöf, Kim L. Gratz, Jonas Bjärehed, Jussi Jokinen, Matthew T. Tull, Brjánn Ljótsson

**Affiliations:** 1 Centre for Psychiatry Research, Department of Clinical Neuroscience, Karolinska Institutet, & Stockholm Health Care Services, Norra Stationsgatan 69, SE-11364 Stockholm, Sweden; 2 Department of Psychology, University of Toledo, Toledo, OH USA; 3 Department of Psychology, Lund University, Lund, Sweden; 4 Department of Clinical Sciences, Psychiatry, Umeå University, Umeå, Sweden; 5 Division of Psychology, Department of Clinical Neuroscience, Karolinska Institutet, Stockholm, Sweden

**Keywords:** Nonsuicidal self-injury disorder, Self-harm, Emotion regulation individual therapy, Emotion regulation, Mediation

## Abstract

**Background:**

Nonsuicidal self-injury (NSSI) is a serious health risk behavior that forms the basis of a tentative diagnosis in DSM-5, NSSI Disorder (NSSID). To date, established treatments specific to NSSI or NSSID are scarce. As a first step in evaluating the feasibility, acceptability, and utility of a novel treatment for adolescents with NSSID, we conducted an open trial of emotion regulation individual therapy for adolescents (ERITA): a 12-week, behavioral treatment aimed at directly targeting both NSSI and its proposed underlying mechanism of emotion regulation difficulties.

**Methods:**

Seventeen girls (aged 13–17; mean = 15.31) with NSSID were enrolled in a study adopting an uncontrolled open trial design with self-report and clinician-rated assessments of NSSI and other self-destructive behaviors, emotion regulation difficulties, borderline personality features, and global functioning administered at pre-treatment, post-treatment, and 6-month follow-up. Measures of NSSI and emotion regulation difficulties were also administered weekly during treatment.

**Results:**

Ratings of treatment credibility and expectancy and the treatment completion rate (88%) were satisfactory, and both therapeutic alliance and treatment attendance were strong. Intent-to-treat analyses revealed significant improvements associated with large effect sizes in past-month NSSI frequency, emotion regulation difficulties, self-destructive behaviors, and global functioning, as well as a medium effect size in past-month NSSI versatility, from pre- to post-treatment. Further, all of these improvements were either maintained or further improved upon at 6-month follow-up. Finally, change in emotion regulation difficulties mediated improvements in NSSI over the course of treatment.

**Conclusions:**

Results suggest the acceptability, feasibility, and utility of this treatment for adolescents with NSSID.

**Trial registration:**

ClinicalTrials.gov (NCT02326012, December 22, 2014, retrospectively registered).

## Background

Nonsuicidal self-injury (NSSI) refers to the deliberate self-inflicted destruction of body tissue (e.g., cutting or burning oneself) without suicidal intent and for purposes not socially sanctioned [1]. NSSI has gained increased scientific attention over the past decade, and was included as a separate diagnostic entity within Section 3 “Conditions for further study” of the Diagnostic and Statistical Manual of Mental Disorders (DSM-5; [2]). Criteria for the suggested NSSI disorder (NSSID) include engagement in NSSI on five or more days within the past year, and the expectation that the behavior will provide emotional or cognitive relief, resolve an interpersonal difficulty, and/or create a positive feeling state.

NSSID is associated with high psychiatric comorbidity (e.g., depressive disorders and anxiety disorders; [3, 4]), and is one of the strongest predictors of future suicide attempts [5, 6]. The prevalence of NSSID in adolescent community samples ranges from 3.1 to 6.7% [7, 8].

Despite the clinical importance of NSSI and NSSID, established treatments specific to NSSI are scarce, and studies evaluating these treatments have produced mixed results [9–12]. In a recent systematic review of treatments for NSSI in both adolescent and adult populations [12], the authors concluded that the psychological treatments that show promise in reducing NSSI frequency are dialectical behavior therapy [13], emotion regulation group therapy (ERGT; [14]), manual-assisted cognitive therapy [15], and dynamic deconstructive psychotherapy [16]. However, another systematic review and meta-analysis on therapeutic interventions for self-injury among adolescents concluded that no psychological interventions are significantly superior to treatment as usual when NSSI is considered separately from suicide attempts [17]. Thus, there is a need for effective treatments for adolescents with NSSI.

ERGT [14] is an acceptance-based behavioral therapy developed to address the need for brief, clinically-feasible, efficacious interventions for NSSI in adults with borderline personality pathology. This 14-week treatment seeks to augment standard therapy provided in the community by directly targeting both NSSI and its proposed underlying mechanism of emotion regulation difficulties. The most recently conducted randomized controlled trial of ERGT for adult women with borderline personality and recurrent NSSI revealed significant effects of this treatment on NSSI and other self-destructive behaviors (e.g., binge-eating and excessive drinking), emotion regulation difficulties, and symptoms of borderline personality disorder (BPD), depression, and anxiety [18]. The utility of ERGT for NSSI has been supported in multiple studies [14, 19, 20]. However, ERGT has not been evaluated for adolescents who engage in NSSI or have NSSID.

Given the efficacy of ERGT for NSSI in adults, as well as its brief and targeted format, we adapted ERGT to provide an ERGT-based individual therapy for adolescents (i.e., Emotion Regulation Individual Therapy for Adolescents; ERITA). Following recommendations for early studies in clinical research [21, 22], the present pilot study examined the feasibility, acceptance, and utility of this ERITA in an open uncontrolled pilot study. We expected that the credibility and acceptability of this treatment would be high. Consistent with past research on ERGT, we also expected to find significant improvements from pre- to post-treatment in NSSI, other self-destructive behaviors, emotion regulation difficulties, and BPD symptoms, as well as stability of these improvements during a 6-month follow up period. Finally, consistent with both past research on ERGT in adults (see [23, 24]) and the theory on which this treatment is based, we expected that change in emotion regulation difficulties would mediate improvements in NSSI during treatment.

## Method

### Design

The present study used an uncontrolled open trial design to examine the acceptability and utility of ERITA for NSSID among adolescent patients in four outpatient clinics in two of the major cities in Sweden, Stockholm and Malmö. All participants were referred from child and adolescent mental health services. Treatment included 12 weekly individual sessions adapted from the ERGT manual. In addition, parents were enrolled in a parent program delivered via the Internet. Baseline data were collected via interviews conducted by community-based health care professionals (with supervision by JB and HS) at the respective clinics and self-report measures. Self-report measures were also administered weekly during treatment. Post assessments were administered directly after the last session and at six months after completing treatment, and included both clinician administered interviews and self-report measures. All self-report measures used in the study were completed online (a method with demonstrated validity; [25]) outside of the clinic.

The study was reported according to the TREND Statement Checklist for nonrandomized interventions [26], and registered on Clinicaltrials.gov (Identifier NCT02326012).

### Participants

Participants included 17 adolescents meeting diagnostic criteria for NSSID. A total of 21 participants were referred to the study and screened for eligibility between June 2014 and April 2015. Eighteen participants met inclusion criteria. Of these, one participant declined participation. All participants provided written informed consent.

Eligibility criteria included: (a) 13–17 years of age; (b) meeting diagnostic criteria for NSSID [2]; (c) having engaged in ≥1 NSSI episode during the past month; (d) having ongoing psychiatric treatment in the community at baseline; (e) having at least one parent who committed to participate in the parent program; and (f) stability of psychotropic medications for at least two months. Exclusion criteria included: (a) a diagnosis of psychotic or bipolar I disorder or ongoing (past month) substance dependence; (b) the presence of co-occurring psychological disorders that required immediate treatment (i.e., severe anorexia nervosa); and (c) insufficient understanding of the Swedish language.

**Fig. 1:**
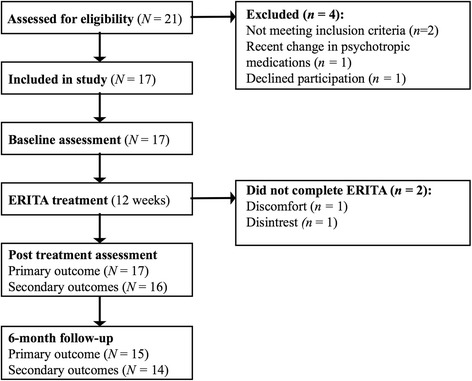
Participant flow through the study

Eighty-eight percent of the enrolled participants met diagnostic criteria for at least one psychological disorder, and the mean length of previous psychological treatment was almost seven months (mean = 6.93; *SD* = 4.46). Diagnostic and demographic data for the final sample are presented in Table 1. Participant flow through the trial is depicted in Fig. 1.

**Table 1:** Sociodemographic, clinical, and diagnostic data of the sample (*N* = 17)

Age (years)	M (SD)	15.31 (1.39)	
	Min-max	13.23–17.66	
Gender	Female	17	100%
Education mother	Primary	0	0%
	Secondary	6	35%
	University	11	65%
Education father	Primary	3	20%
	Secondary	6	40%
	University	6	40%
Ongoing psychotropic medication		5	29%
Earlier psychological treatments	Yes	14	82%
	Mean length in months (*SD)*	6.93 (4.46)	
Meeting full diagnostic criteria for BPD	Yes	7	41%
Mean number of BPD criteria		3.82 (1.42)	
Number of participants with suicidal behavior, lifetime	Actual attempt	4	24%
	Interrupted attempt	4	24%
	Aborted attempt	2	12%
	Preparatory acts	2	12%
NSSI frequency past 12 months	Median	110	
	min - max	8–390	
Frequency of co-occurring disorders	Depression	7	41%
	Panic disorder	7	41%
	ADHD	9	53%
	Conduct disorder	4	24%
	Oppositional deficient disorder	6	35%
	Social anxiety disorder	5	29%
	Posttraumatic stress disorder	2	12%
	Separation anxiety	2	12%
Number of participants with 0–5 co-occurring disorders	None	2	12%
	One	3	18%
	Two	5	29%
	Three	1	6%
	Four	4	24%
	Five	2	12%

*Note.* ADHD = Attention deficit hyperactivity disorder, BPD = Borderline personality disorder, NSSI = Nonsuicidal self-injury

### Measures

#### Diagnostic assessments

Diagnostic assessments were conducted by community-based health care professionals. Presence of NSSID was assessed using the Clinician-Administered Nonsuicidal Self-Injury Disorder Index [27]. To determine if participants met criteria for DSM-IV Axis I disorders, the MINI-KID International Neurospychiatric Interview version 6 [28] was used. Finally, BPD symptoms were assessed using the Structured Clinical Interview for DSM-IV Personality Disorders BPD Module [29]. The results from these DSM-IV-based interviews are presented in DSM-5 nomenclature, as the essential features of the majority of disorders assessed in this study remained the same from DSM-IV to DSM-5. One exception to this is posttraumatic stress disorder, for which the diagnostic thresholds for children and adolescents were lowered in DSM-5; thus, it is possible that the assessment of DSM-IV posttraumatic stress disorder may have resulted in lower rates of this disorder relative to the use of DSM-5 criteria.

#### Acceptability measures

The Credibility/Expectancy Scales [30] were used to assess the perceived credibility of ERITA on a 9-point scale and patients’ expectancies regarding its benefits on a 11-point scale. Evidence for the reliability and predictive validity of this measure has been provided [31]. Higher scores indicate greater credibility (range 1–9) and expectancy (range 0–100%). This measure was administered at the end of the first therapy session. The Working Alliance Inventory – Short Form (WAI-S; [32]) is a 12-item scale that measures aspects of the therapeutic alliance, including agreement on tasks and goals of the ongoing therapy and the development of an affective bond between the therapist and the patient. Items are rated on a 7-point scale ranging from *Never* to *Always*. In this study, we used a revised 6-item version of this measure (WAI-SR; [33]). The WAI-SR was administered after session three.

#### Primary outcome measure

The 9-item Deliberate Self Harm Inventory (DSHI-9; [34]) was used to assess the primary outcome of NSSI frequency. The DSHI-9 is a modified version of the DSHI [35], an empirically-supported measure of various aspects of NSSI originally developed for use with adults. This 9-item measure, adapted for use with adolescents, assesses the presence and frequency of the most common forms of NSSI in adolescents, including cutting, burning, severe scratching, self-biting, carving, sticking sharp objects into the skin, self-punching, and head banging (all to the extent that scarring, bleeding, and/or bruising occurred). Although this measure also assesses preventing wounds from healing, this item was omitted from all analyses, as picking a scab is considered to be a normative and less severe behavior and is often not categorized as NSSI [2]. The DSHI-9 has demonstrated good test-retest reliability and adequate concurrent validity among adolescents [34]. The DSHI-9 was also used to calculate NSSI versatility (i.e., the number of different types of NSSI behaviors in the past month), which has been shown to be an indicator of NSSI severity [36]. The DSHI-9 was administered at pre-treatment, post-treatment, and 6-month follow-up to assess past month engagement in NSSI. The DSHI-9 was also administered every week during treatment to assess past week engagement in NSSI.

#### Secondary outcome measures

The Difficulties in Emotion Regulation Scale (DERS; [37]) is a 36- item self-report measure that assesses individuals’ typical levels of emotion regulation difficulties on a 5-point Likert-type scale across six domains: nonacceptance of negative emotions, inability to engage in goal-directed behaviors when distressed, difficulties controlling impulsive behaviors when distressed, limited access to emotion regulation strategies perceived as effective, lack of emotional awareness, and lack of emotional clarity. The DERS has been found to demonstrate good reliability and construct and convergent validity in both adult [37, 38] and adolescent [39, 40] samples*.* The DERS was administered at pre-treatment, post-treatment, and 6-month follow-up. Internal consistency in this sample was acceptable (α = .85).

A brief, 16-item version of the DERS, the DERS-16 [41], was administered weekly during treatment. The DERS-16 has demonstrated good test-retest reliability and construct and predictive validity [41, 42]. Internal consistency in this sample was acceptable (α = .79).

The 11-item behavior supplement to the Borderline Symptom List (BSL; [43]) measures engagement in a variety of impulsive, self-destructive behaviors (e.g., risky sexual behavior, binge eating, and substance abuse). Items are rated on a 5-point Likert-type scale ranging from *Not at all* to *Daily or more often* and a total score is calculated by summing all items (e.g., [19]). The BSL-supplement was administered weekly during treatment, as well as at pre-treatment, post-treatment, and 6-month follow-up.

The Borderline Personality Feature Scale for Children (BPFS-C; [44]) is a 24–item questionnaire that assesses BPD features in youth ages nine and older. It includes items assessing the following four subscales: Affective Instability, Identity Problems, Negative Relationships, and Self-harm. Participants rate each item using a 5-point scale ranging from 1 (not at all true) to 5 (always true). Evidence for the internal consistency and convergent and criterion validity of the BPFS-C in both clinical and nonclinical samples of youth has been provided [44–46]. The BPFSC was administered at pre-treatment, post-treatment, and 6-month follow-up. Internal consistency in this sample was acceptable (α = .73).

#### Clinician-rated outcome measure

The Children’s Global Assessment Scale (CGAS; [47]) was used to assess global functioning. The CGAS ranges from 0 to 100, with higher scores indicating better functioning. The CGAS has shown moderate to excellent inter-rater reliability, good stability over time, and good concurrent and discriminant validity [47–49]. The CGAS was administered at pre-treatment, post-treatment, and 6-month follow-up.

### Treatment

ERITA is an ERGT-based individual therapy for adolescents adapted from the original ERGT treatment manual used in previous studies of adult women with NSSI (e.g., [19, 20, 50]). The primary adaptations to ERGT included: 1) providing the treatment in an individual versus group format (to avoid any iatrogenic effects related to social contagion; e.g., [51]); 2) shortening the treatment to 12 weeks so that it could be provided over the course of one school semester and including a final session on relapse prevention (which was accomplished by combining the two sessions focused on increasing emotional clarity into one session and the four sessions focused on valued directions and engagement in valued actions into two sessions); 3) simplifying the homework sheets; 4) incorporating a youth-friendly design and format and age-appropriate examples; and 5) including an Internet-delivered course with online therapist support for parents (in line with past research on family support in the treatment of adolescents with self-harming behaviors; e.g. [52]). Table 2 provides an overview of the structure and specific topics addressed in ERITA each week.

**Table 2:** An overview of the content of the ERITA treatment protocol

Adolescent treatment	Parent program
(Module) Content	(Module) Content
(1) Functions of NSSI Information about NSSI, exploring the functions driving NSSI and other self-destructive behaviors. Introduction to the concept of valued direction.	(1) Psychoeducation Information about NSSI, emotional reactivity, the role of an invalidating environment, and skills in validation.
(2) Functionality of emotions Facts about basic emotions, the functionality of emotions and why it is important to be aware of them.	(2) Repetition and homework review
(3) Emotional awareness Increase emotional awareness by becoming aware of the different components of emotions.	(3) How to improve parenting in the long run Activate yourself and your child in order to increase positive interactions.
(4) Emotions provide information that can guide our behavior How to identify the information that emotions provide about our environment and ourselves. How to act on that information or express emotions in healthy ways.	(4) Repetition and homework review
(5) Primary vs. secondary emotions Introducing the concept of primary and secondary emotions, how to differentiate between them, and why this is important.	(5) Conflict management and problem solving Emotional awareness and six steps for problem solving.
(6) Emotional avoidance / unwillingness Conceptualize problematic behaviors as an act of unwillingness. Metaphors describing the paradoxical consequences of our efforts to control/suppress emotions.	(6) Repetition and homework review
(7) Emotional willingness / acceptance Willingness as the solution. What willingness is and is not.	(7) Summary and evaluation Repetition and evaluation.
(8) Non-avoidant emotion regulation strategies How to regulate emotions by distraction and approach strategies.	
(9) Take control over impulsive behaviors Definition of impulsive behaviors. Techniques for resisting urges: distraction, behavior substitution, and remind yourself of long-term negative consequences, and consequence modification.	
(10) Valued directions Values are different from goals. Identify valued directions.	
(11) Commitment to valued actions Identify and commit to valued actions.	
(12) Relapse prevention Identify which skills have been helpful; identify high risk situations and strategies for preventing relapse.	

The Internet-based, adjunctive parent program was developed specifically to augment ERITA and consisted of four main modules and three follow-up modules that paralleled the individual therapy sessions of ERITA. The online program combined psycho-education with interactive exercises. The modules addressed specific parent-related topics, such as attitudes toward NSSI and other self-destructive behaviors, effective communication skills (e.g., validation), strategies to increase activities and interactions with the adolescent that are not focused on mental health problems, conflict management, and problem solving. An outline of the structure and topics addressed in the parent program is presented in Table 2. In addition to their own material, the parents also had access to the blank worksheets from the adolescents’ treatment (i.e., the psychoeducation and exercises) so that they were aware of the skills their children were learning. During the 12-week treatment period, parents had regular online therapist support to help problem solve, guide them through the program, and help with the youth’s homework assignments when necessary.

### Therapist training and treatment adherence

Four therapists experienced in treating youth with NSSI delivered ERITA. Prior to the study, all therapists familiarized themselves with the treatment manuals of both ERGT and ERITA. In addition, all therapists participated in a three-day workshop led by the authors of the ERGT manual (KLG and MTT). Further training in ERITA was provided by the authors of its manual (JB and HS). To ensure therapist treatment adherence, all sessions were filmed and all therapists received the option of weekly supervision based on the reviewed films by the authors of the ERITA manual (JB and HS). Finally, the online therapist support provided to parents was delivered by JB and HS.

### Data analysis

Statistical analyses were performed using R version 3.3.1 [53]. Primary analyses used a generalized estimation equation (GEE) with an exchangeable working correlation along with robust error estimation. The distribution of each outcome was examined prior to the analyses. The GEE models for the count variables (i.e., NSSI frequency and self-destructive behaviors) used a negative binomial distribution with a log link function, and the remaining continuous outcomes were analyzed using GEE models with a normal distribution with a log link function. Consistent with an intent-to-treat principle, all models included all available data for each outcome. We estimated separate coefficients for the change between the pre- and post-treatment assessments and the change between the post-treatment and six-month follow-up assessments. Regression weights that were inversely related to the probability of a value being observed as a function of time were included in the GEE models (an approach that gives unbiased estimation under the assumption of data missing at random; [54, 55]). Ordinal variables were analyzed using Wilcoxon signed ranks test.

Effect sizes were calculated for changes between pre-treatment and post-treatment, and post-treatment and 6-month follow-up. First, the average percentage change across time for the count variables (i.e., NSSI frequency and BSL) was calculated from the GEE models with 95% confidence intervals. This was performed by exponentiating the relevant beta, with the range below or above one interpreted as the percentage decrease or increase in the outcome for a one-unit increase in the predictor. Second, Cohen’s *d* was calculated for the remaining continuous outcomes by dividing the estimated means derived from the GEE models by the baseline standard deviation. For comparative reasons (with other studies), Cohen’s *d* was also calculated for the count data, for which the GEE models used a normal distribution with log link function that were applied to log-transformed NSSI frequency and BSL scores, and corresponding effect sizes were extracted (these analyses were only used for effect size estimation, not to draw inferences about statistical significance or percentage change). The 95% confidence intervals for the effect sizes were calculated using 5000 bootstrap replications [56] clustered on participants; i.e., if one participant was included in a bootstrap replication, all observations from this participant were included in the bootstrap replication [57]. Finally, we calculated the number of participants who reported no (zero) NSSI at pre-treatment, post-treatment, and 6-month follow-up and used McNemar’s mid-*p* test [58] to analyze the changes between the assessment points (two observations were missing from the 6-month follow-up, and list-wise deletion was used in these specific analyses).

We also examined change in emotion regulation difficulties as a mediator of improvements in NSSI during treatment. Specifically, this analysis was performed in four steps that investigated the different paths of mediation [59]: (1) the “*c*-path”, i.e., if week in treatment was associated with NSSI frequency; (2) the “*a*-path”, i.e., if week in treatment was associated with emotion regulation difficulties; (3) the “*b*-path”, i.e., if emotion regulation difficulties were associated with NSSI frequency (controlling for week in treatment); and (4) the *ab*-product, i.e., the indirect relation of time in treatment to improvement in NSSI through change in emotion regulation difficulties. We also calculated the proportion of the total relation of time in treatment to NSSI improvement that was accounted for by change in emotion regulation, P_M_, using the formula *ab* / *c* [60]. These analyses were performed using linear mixed effects models with random intercepts and slopes; NSSI frequency was log-transformed to fit the linear model.

Finally, we performed exploratory supplementary analyses to investigate the possible impact of concurrent medication use and treatment as usual on NSSI improvement during treatment. We did this by adding self-reported concurrent medication status (coded as 0 for no concurrent medication and 1 for concurrent medication) and self-reported frequency of contact with community clinicians (coded as a factor with the following levels: no contact during ERITA, monthly, once every second week, or weekly) as covariates in the model. These covariates were added both as simple effects and as interaction effects with the change in NSSI frequency from pre- to post-treatment.

## Results

### Treatment adherence, attrition, and acceptability

Of the 17 participants who began ERITA, two (12%) dropped out of treatment after session 2 (see Fig. 1). Reported reasons for dropping out included discomfort with and disinterest in the treatment and its format. The average number of treatment sessions attended for all included participants was 10.29 (*SD* = 3.37; median = 12) out of 12 sessions. All enrolled participants completed post-treatment assessments and 88% (*n* = 15) completed 6-month follow-up assessments. Mean ratings of treatment credibility (*M* = 6.14, *SD* = 2.07) and expectancy (*M* = 56.43%, *SD* = 22.74) completed after the first session were satisfactory and comparable to findings from previous ERGT studies (e.g. [19, 20]). Participants also rated their alliance with their therapist (on the WAI-SR) as high (*M* = 32.15, *SD* = 9.90).

Although all participants were required to have some form of ongoing psychiatric treatment in the community when they enrolled in the study, 24% of participants (*n* = 4) reported at post-treatment that they had not met with their other treatment provider during the 12 weeks of ERITA, 59% (*n* = 10) reported only monthly meetings with their community provider, and 6% (*n* = 1) reported twice monthly meetings with their community provider. The corresponding figures at the 6-month follow-up were 18%, 35%, and 29%. At post-treatment, 94% of participants (*n* = 16) stated that ERITA had been their primary treatment during the past twelve weeks.

### Primary analyses

Medians, inter quartile range, means, standard deviations, percentage change, and Cohen’s *d* for all outcome measures are presented in Table 3.

**Table 3:** Treatment outcome variables at pre-treatment, post-treatment and 6-month follow-up

Outcome	Pre-treatment	Post-treatment	6-mo f-u	Pre-to post-treatment comparison		Post- to 6-mo follow-up comparison		Pre-to 6-mo follow-up comparison	
*Count- data*	*Median (IQR)*	*Median (IQR)*	*Median (IQR)*	*Z*	*Percentage change [95% CI]*	*Z*	*Percentage change [95% CI]*	*Z*	*Percentage change [95% CI]*
DSHI-9-f	8 (4 to 13)	2.5 (0 to 4.25)	0 (0 to 3)	2.33*	42 [8 to 64]	3.82***	63 [39 to 78]	4.77***	79 [60 to 89]
BSL	3 (2 to 3)	1 (0 to 3)	1 (0 to 2.75)	2.16*	44 [5 to 67]	0.35	13 [−91 to 60]	1.99*	52 [1 to 76]
*Continuous*	*Mean (SD)*	*Mean (SD)*	*Mean (SD)*	*Z*	*Cohen’s d [95% CI]*	*Z*	*Cohen’s d [95% CI]*	*Z*	*Cohen’s d [95% CI]*
DSHI-9-v	2.12 (1.54)	1.35 (1.22)	0.80 (1.01)	2.02*	0.50 [0.07 to 0.78]	2.24*	0.40 [0.09 to 0.80]	2.61**	0.89 [0.47 to 1.23]
DERS	122.71 (17.18)	109.69 (25.21)	100.57 (17.89)	2.91**	0.81 [0.23 to 1.50]	2.66**	0.59 [0.15 to 1.05]	4.44***	1.40 [0.87 to 2.03]
BPFSC	70.35 (10.72)	66.88 (12.61)	63.36 (11.34)	1.45	0.33 [−0.03 to 1.11]	1.47	0.36 [−0.16 to 0.87]	2.73**	0.69 [0.18 to 1.19]
CGAS	51.24 (4.94)	56.59 (7.97)	61.07 (11.21)	3.7***	1.08 [0.51 to 1.62]	1.72	0.85 [−0.22 to 2.13]	4.74***	1.94 [1.03 to 3.12]

*Note.* Count data. Test statistics are based on generalized estimation equation models using either a negative binomial or normal distribution with log link function for count and continuous data, respectively. Effect sizes for count data were based on log-transformed scores. Confidence intervals for effect sizes are based on 5000 bootstrap replications. Abbreviations: BPFSC = Borderline Personality Feature Scale for Children, BSL = Borderline Symptom List, CGAS = Children’s Global Assessment Scale, DERS = Difficulties in Emotion Regulation Scale, DSHI-9-f = Deliberate Self-Harm Inventory – frequency past month, DSHI-9-v = Deliberate Self-Harm Inventory – versatility past month. *p < .05, **p < .01, ***p < .001

#### Primary outcomes

A significant large reduction in past month NSSI frequency (*d* = 1.14, 95% CI: 0.60, 1.95) was found from pre- to post-treatment. Additionally, results revealed further significant improvements in NSSI frequency (*d* = 0.76, 95% CI: 0.27, 1.41) from post-treatment to 6-month follow-up. The observed means for NSSI frequency were 9.53 (*SD* = 7.76), 5.47 (*SD* = 8.15), and 2.20 (*SD* = 3.30) at pre-treatment, post-treatment, and follow-up, respectively. Likewise, results revealed a significant reduction in past-month NSSI versatility associated with a medium-sized effect from pre- to post-treatment, with additional significant improvements in NSSI versatility from post-treatment to 6-month follow-up. The most common past-month NSSI method at pre-treatment was cutting (*n =* 17; 100%), followed by severe scratching (*n =* 7; 41%), skin carving (*n =* 3; 18%), needle-sticking (*n =* 3; 18%), self-punching (*n =* 2; 11%), head-banging (*n =* 2; 11%), burning (*n =* 1; 6%), and self-biting (*n* = 1; 6%). The corresponding numbers for post-treatment and six-month follow-up were *n* = 11 (65%) and *n* = 6 (40%) for cutting, *n* = 5 (29%) and *n* = 3 (20%) for severe scratching, *n* = 1 (6%) and *n* = 0 for skin carving, *n* = 1 (6%) and *n* = 0 for needle-sticking, *n* = 4 (24%) and *n* = 2 (13%) for self-punching, *n* = 1 (6%) and *n* = 0 for head-banging, *n* = 0 and *n* = 0 for burning, and *n* = 0 and *n* = 1 (7%) for self-biting, respectively. Finally, the proportion of participants with past month NSSI abstinence increased significantly (*p* = .031) from 0% at pre-treatment to 29% at post-treatment, and remained stable (*p* = .063) from post-treatment to 6-month follow up (53%).

#### Secondary outcomes

Results revealed significant improvements in emotion regulation difficulties, number of self-destructive behaviors (*d* = 0.82, 95% CI: 0.23, 1.81), and global functioning (associated with large effect sizes) from pre- to post-treatment, and further significant improvements in emotion regulation difficulties during the follow up period. There were no significant changes in self-destructive behaviors (*d* = 0.09, 95% CI: -0.95, 0.82) or global functioning during the follow-up period. The observed means for BSL were 3.00 (*SD* = 2.21), 1.69 (*SD* = 2.06), and 1.50 (*SD* = 1.79) at pre-treatment, post-treatment, and 6-month follow-up, respectively.

Finally, symptoms of BPD decreased but did not improve significantly from pre-treatment to post-treatment or from post-treatment to 6-month follow-up. However, the improvement in BPD symptoms from pre-treatment to 6-month follow up was significant.

#### Weekly measures

**Fig. 2:**
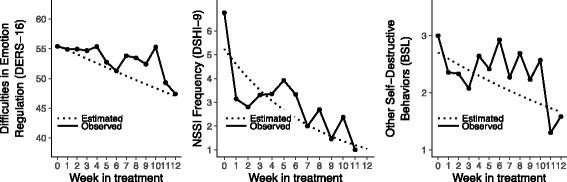
Observed and estimated scores show a significant decrease in difficulties in emotion regulation (*P* = .028), NSSI frequency (*P* < .001), and other self-destructive behavior (*P* < .001)

Figure 2 shows the weekly means for NSSI frequency (on the DSHI-9), number of other self-destructive behaviors (on the BSL), and emotion regulation difficulties (on the DERS-16) during treatment, together with estimated regression lines for each outcome measure. The GEE analyses revealed significant time effects for NSSI frequency (Z = 4.68, *p* < .001), other self-destructive behaviors (Z = 1.30, *p* < .001), and emotion regulation difficulties (Z = 2.19, *p* = .028).

#### Mediation analysis

Consistent with hypotheses, the a-path (estimate = −0.85, SE = 0.34, *p* = .013), b-path (estimate = 0.02, SE = 0.01, *p* = .006), and c-path (estimate = −0.58, SE = 0.02, *p* = .006) were all significant, revealing significant relations between time in treatment and improvements in both NSSI and emotion regulation difficulties, as well as a significant relation between emotion regulation and NSSI improvements during treatment. Finally, the indirect relation of time in treatment to NSSI improvement through change in emotion regulation (a x b) was also significant (estimate = −0.02, SE = 0.01, *p* = .046), with change in emotion regulation difficulties accounting for 32% of the improvements in NSSI frequency.

#### Supplementary analyses

Results of the analyses including concurrent medication status and community clinician contact as simple effects and interaction effects with the change in NSSI frequency from pre- to post-treatment revealed that none of the added predictors were statistically significant (*p* > .05), suggesting that neither concurrent medication use nor ongoing clinical contact was associated with improvement in NSSI frequency during treatment.

## Discussion

The present study provides preliminary support for the feasibility, acceptability, and utility of ERITA in the treatment of NSSI and related difficulties among adolescents with NSSID. Results revealed significant improvements associated with medium to large effect sizes in past-month NSSI frequency, past-month NSSI versatility, emotion regulation difficulties, self-destructive behaviors, and global functioning from pre- to post-treatment. Further, all of these improvements were either maintained or further improved upon at 6-month follow-up. Moreover, by the 6-month follow-up (although not before), symptoms of BPD had improved significantly from pre-treatment levels. Finally, the high ratings of treatment credibility and expectancy, combined with the good attendance and low attrition, highlight the acceptability and feasibility of this treatment among adolescents. Although preliminary, these findings are encouraging and comparable to those of past studies examining brief treatments for NSSI and/or self-injurious behaviors in adolescents and young adults [61–65].

Furthermore, and consistent with past research on ERGT [23], results of the present study provide support for emotion regulation as a mechanism of change in ERITA. Specifically, change in emotion regulation difficulties mediated the observed improvements in NSSI during treatment. These results provide support for the theoretical model underlying this treatment and add to the literature emphasizing the clinical utility of targeting emotion regulation in treatments for NSSI (see [66, 67]).

Notably, inclusion criteria for this study were broad and exclusion criteria were kept to a minimum in order to enhance ecological validity. Although we did not explicitly recruit adolescents with BPD, nearly half of the sample (41%) met full DSM-IV criteria for BPD (consistent with past research suggesting high rates of BPD in clinical samples of adolescents with NSSI; e.g., [68, 69]. Findings of small, non-significant improvements in BPD symptoms from pre- to post-treatment suggest that this treatment is not sufficient to have a meaningful impact on BPD symptoms beyond NSSI and other self-destructive behaviors in just 12 weeks. Rather, findings of a significant improvement in BPD symptoms from pre-treatment to the 6-month follow up suggest that a longer period of time may be necessary for the non-behavioral symptoms of BPD to change. It should be noted, however, that the risk for type II error due to low power was relatively high in this study. Therefore, replication of these findings in larger samples is needed before conclusions regarding the impact of this treatment on BPD symptoms can be drawn.

This study is the first to evaluate an adapted version of ERGT for adolescents. Following recommendations for early evaluations of treatments [21, 22], the utility of this treatment was examined in an uncontrolled open pilot design. There are several limitations that warrant discussion, some of which are inherent to uncontrolled pilot studies. Most importantly, the absence of a randomized controlled design and/or control condition precludes any conclusions regarding the effects of ERITA (vs. the passage of time) on the outcomes of interest. Likewise, because all participants were required to have some form of psychiatric treatment in the community in addition to ERITA at baseline (and 29% reported concurrent psychotropic medication use), the potential impact of these treatments on the observed improvements cannot be determined. Nonetheless, it is important to note that the vast majority of the participants in this study (83%) reported minimal contact with their community provider over the course of ERITA, and our supplementary analyses of the impact of concurrent medication usage and community provider contact on reductions in NSSI frequency revealed no significant relations between these concurrent treatments and changes in NSSI over the course of treatment. Thus, it is unlikely that the observed improvements in NSSI in this study were the result of these community treatments alone. These results also highlight the potential utility of ERITA as a stand alone treatment for NSSI in adolescents. Further research examining the efficacy of this treatment in a randomized controlled trial is needed to further establish its utility for adolescents with NSSID.

An additional limitation of this study is the relatively small sample size, which reduces both our statistical power and the generalizability of our findings. Indeed, sufficient participant recruitment (in terms of both the breadth and size of the sample) is a critical component of any clinical trial and has important implications for interpreting study results. Yet, little is known about effective recruitment strategies for at-risk clinical populations, such as adolescents with NSSID, and there is almost no information available to researchers to aid in planning effective recruitment procedures in treatment outcome studies. For example, despite working closely with Child and Adolescent Psychiatry, we were only able to recruit 17 participants in an 8-month period. Future research is needed to explore possible barriers to more effective and efficient recruitment, including lack of knowledge of novel interventions, sufficient access to and satisfaction with available treatments in the community, lack of interest in or concern about clinical trials, or clinician reluctance to refer patients to a treatment study. Likewise, although repeated NSSI is also reported among boys [7, 8], male gender has been shown to increase the likelihood of not receiving treatment after self-injury [70]. Consistent with this research, and despite no gender-based inclusion or exclusion criteria in this study, the final sample consisted of only girls. Thus, the extent to which the results generalize to boys is unclear and additional research examining the utility of this treatment in larger, mixed-gender samples is needed.

## Conclusions

Results provide preliminary support for the acceptability, feasibility, and utility of ERITA for adolescents with NSSID. Given the large within-group effect sizes found in this open trial, a randomized controlled trial studying treatment efficacy is called for.

## Abbreviations

ADHD: Attention deficit hyperactivity disorder
BPD: Borderline personality disorder
BPFS-C: Borderline Personality Feature Scale for Children
BSL: Borderline Symptom List
CGAS: Children’s Global Assessment Scale
CI: Confidence interval
DERS: Difficulties in Emotion Regulation Scale
DSHI: Deliberate self-harm inventory
DSM: Diagnostic and Statistical Manual of Mental Disorders
ERGT: Emotion regulation group therapy
ERITA: Emotion regulation individual therapy
GEE: Generalized estimation equation
NSSI: Nonsuicidal self-injury
NSSID: Nonsuicidal self-injury disorder
WAI-S: Working Alliance Inventory – Short Form
